# Parayangiella, A New Genus of Mezirinae from China (Hemiptera: Aradidae) †

**DOI:** 10.3390/insects16111121

**Published:** 2025-11-01

**Authors:** Tianci Zhang, Wanzhi Cai, Ernst Heiss, Xiaoshuan Bai

**Affiliations:** 1 College of Life Science and Technology, Inner Mongolia Normal University, Hohhot 010022, China; ztc1112025@126.com; 2 Department of Entomology, China Agricultural University, Yuanmingyuan West Road, Beijing 100193, China; caiwz@cau.edu.cn; 3 Tiroler Landesmuseum, Josef-Schraffl-Strasse 2a, A-6020 Innsbruck, Austria

**Keywords:** Aradidae, new species, phylogenetic analysis, mitochondrial genome, taxonomy

## Abstract

This paper describes a new genus of Aradidae from China: *Parayangiella* gen. n., with its type species *Parayangiella latiovatusa* sp. n. Their diagnostic morphological characteristics are presented with illustrations, and molecular analyses are conducted. In addition, based on the sequence data of 13 protein-coding genes (PCGs), this study employed two methods—maximum likelihood (ML) and Bayesian inference (BI)—to construct the phylogenetic tree. The results are as follows: The mitochondrial genome of *Parayangiella latiovatusa* is a closed circular double-stranded structure, consisting of 13 protein-coding genes, a control region, 22 tRNA genes and 2 rRNA genes, which is consistent with most species in the family Aradidae. The mitochondrial genome of *Parayangiella latiovatusa* sp. n. exhibits AT skew. The phylogenetic tree construction results show that *Parayangiella latiovatusa* sp. n. and the genus *Yangiella* form a sister group.

## Simple Summary

Aradidae is recognized as a large family belonging to the order Hemiptera. Its members primarily inhabit the underbark of decaying wood, and often live in groups. Most of them feed on the mycelia of rotting wood. In recent years, the mitochondrial genome has become an important molecular marker in insect taxonomy and phylogeny. In this study, a detailed morphological description and molecular analysis were conducted on *Parayangiella latiovatusa* sp. n., the type species of a new genus collected from Yunnan (China). Furthermore, through the phylogenetic analysis conducted on mitochondrial genome data, the taxonomic status of the genus *Parayangiella* was discussed.

## 1. Introduction

Mezirinae is the largest subfamily of Aradidaea and has a widespread global distribution, including over 57 genera in the Oriental region [1]. Among them, three genera, *Pseudomezira* Heiss, 1982 from China, *Neartabanus* Heiss, 1999 from Laos and India, *Longitergus* Pham, Bai, Heiss & Cai, 2013 from Vietnam, are macropterous; four genera, *Brachybarcinus* Heiss, 2010 from China and Vietnam, *Hainanmezira* Bai, Heiss & Cai, 2011 from China, *Stipesoculus* Bai, Wu & Cai, 2007 from China, and *Pseudowuiessa* Shi, Bai, Wu, Heiss & Cai, 2016 from China, are brachypterous [2,3,4,5,6,7,8,9,10]. In studies on the flat bug family Aradidae in China, we discovered a remarkable species that is morphologically similar to *Neuroctenus* Kormilev, 1980 and *Yangiella* Liu, 1980 but no known genus within Mezirinae can accommodate this species [11,12]. Consequently, we propose a new genus *Parayangiella* gen. n. to encompass this species.

Mitochondrial genomes serve as one of the core sources of molecular markers in animal phylogeny studies [13]. Molecular studies on Aradidae include: Ji et al. (2024) conducted phylogenetic studies on three *Yangiella* species, with the determination of their mitochondrial genomes as a key step [14]; and Li et al. (2025) sequenced the complete mtDNA genome of *Neuroctenus hainanensis* [15]. In this study, the newly discovered *Parayangiella latiovatusa* sp. n. was investigated through an integration of traditional morphology and mitochondrial genomics.

## 2. Materials and Methods

### 2.1. Specimens and Taxonomy

The holotype is deposited in the Museum of Biological Specimens, Inner Mongolia Normal University, Hohhot, China. Specimens were observed under a Motic SMZ-168 Series microscope and documented using a Keyence VH-S30B digital microscope. Morphological terminology follows Heiss [16] and Bai et al. [17].

### 2.2. Gene Assembly, Annotation, and Analysis

To obtain genomic DNA for subsequent analyses, it was extracted from the thoracic muscle of a single specimen of *Parayangiella latiovatusa* sp. n., using a kit from Tiangen Biochemical Technology Co., Ltd. (Beijing, China). Subsequently, the extracted DNA—with a concentration of ≥20 ng/µL and a total amount of ≥500 ng—was sent to Beijing Berry Genomics Co., Ltd. (Beijing, China) for sequencing. Sequencing libraries were constructed from individual insect specimens and subjected to paired-end sequencing with 150 bp read lengths on the Illumina NovaSeq 6000 platform (S4 flow cell, PE150 mode). Following quality control by the company, approximately 6 GB of clean data were obtained.

Assembly was performed using SPAdes v3.15.5 (https://github.com/ablab/spades (accessed on 15 April 2025)) [18]. Based on the contig sequences obtained through an iterative method during the assembly process, a local blast database was constructed. The cox1 sequence of the closely related species *Neuroctenus yunnanensis* (NC 063144) was used as a bait to screen the target mitochondrial genome contigs using the BLAST v2.15.0 function. The sequence was uploaded to MITOS [19] (http://mitos.bioinf.uni-leipzig.de/index.py (accessed on 23 April 2025)) for subsequent annotation, with the settings adjusted to the invertebrate mitochondrial genetic code. After importing the annotation results into Geneous Prime v11 software [20], using the gene boundaries of closely related species as a reference, the gene boundaries of the target species were manually corrected. The mitochondrial genome of *Parayangiella latiovatusa* sp. n. has been submitted to GenBank with the accession number PP737162.

The CGView Server (https://cgview.ca/ (accessed on 26 May 2025)) [21] was utilized to generate the circular map of the complete mitochondrial genome. MEGA11.0 was employed to calculate the nucleotide composition of the gene [22]. RSCU (relative synonymous codon usage) was analyzed using CodonW v1.4.4. The Ka (non-synonymous) and Ks (synonymous) substitution rates of PCGs were computed using DnaSP v6.12.03 [23], and the results were visualized in R. Substitution saturation was assessed using DAMBE v7.3.32 [24].

### 2.3. Phylogenetic Analyses

Mitochondrial genomes of 13 insect species were retrieved from NCBI, including 12 species of Aradidae, with *Pentatoma metallifera* and *Epidaus famulus* as outgroups (Table A1). Combined with the mitochondrial genome of the *Parayangiella* species, an initial dataset was constructed. To extract PCG nucleotide sequences, we imported the raw dataset into PhyloSuite v1.2.3 [25]. Subsequently, MAFFT v7.464 software [26] was used to perform multiple sequence alignment of the PCG, and the aligned sequences were then processed using MACSE [27] for optimization, thereby enabling accurate identification of pseudogenization events. Thereafter, GBlocks v0.91b [28] was employed to trim the PCGs, followed by concatenation using FASconCAT-G v1.04 [29] to generate the final dataset.

Model Finder [30] was used to partition the concatenated datasets, after which model selection was conducted to determine the most appropriate evolutionary model. Based on the results from Model Finder (Table A2 and Table A3), Exabayes v1.5.1 software [31] was employed to construct a BI (Bayesian inference) phylogenetic tree. The Bayesian inference used 10,000,000 MCMC generations (sampling every 1000th, 25% burn-in). A Bayesian phylogenetic tree was generated after convergence (ASDSF < 0.01). Then, a Maximum Likelihood (ML) phylogenetic tree was constructed using IQ-tree v2 software [32] with 100,000 ultrafast bootstrap replicates [33]. Finally, the tree files were imported into the online server TVBOT (https://www.chiplot.online/tvbot.html (accessed on 12 July 2025)) [34] for phylogenetic tree beautification.

## 3. Results

### 3.1. Taxonomy

Aradidae Brullé, 1836 [35]

Mezirinae Oshanin, 1908:478 (Mezirina, as subfamily) [36]. Type genus: *Mezira* Amyot & Serville, 1843 [37].

#### 3.1.1. *Parayangiella* Bai, Cai & Heiss, gen. N

urn:lsid:zoobank.org:pub:73A12CC8-B2ED-451E-9770-ACA200514B15

 

Type species: *Parayangiella latiovatusa* Bai, Cai & Heiss, sp. n.

Diagnosis. Although morphologically similar to *Neuroctenus* and *Yangiella*, this taxon cannot be placed in any known genus of Mezirinae; here therefore *Parayangiella* gen.n. is proposed. It is distinguished from *Neuroctenus* by its ventrally convex body (versus flattened), the absence of a transverse carina at the base of abdominal sternites IV–VI (versus present or replaced by longitudinal folds), and its broadly widened abdomen (versus not broadly widened). In contrast to *Yangiella*, *Parayangiella* can be identified by its anteriorly angularly produced pronotum (versus not angularly produced), non-parallel body sides with a broadened posterior end (versus nearly parallel sides without broadening), and the presence of smooth protuberances on either side of the male sternite VII (versus absent).

Key to related genera

1 Body flattened; abdominal sternites 4–6 each with a transverse carina at base; if carina indistinct, sternites with sublateral longitudinal folds .................... *Neuroctenus* Kormilev
- Body ventrally convex; abdominal sternites 4–6 without transverse carina at base .......................................................................................................... . ......................................... 2
2 Body sides nearly parallel, posterior end not broadened; male sternite 7 without smooth protuberances on either side of center ............. ........ ....................................... *Yangiella* Liu
- Body sides not parallel, posterior end broadened; male sternite 7 with smooth protuberances on either side of center ............... .. ...... ........ *Parayangiella* Bai, Cai & Heiss, gen. n.

Description. Body coloration: brown to black; eyes red; hemelytral membrane black. Macropterous. Surface: granulate on head, antennae, pronotum, scutellum, and legs.

Head. Anterior process short, reaching or slightly exceeding apex of antennal segment I. Antennae: 4-segmented; segment I clavate; segments II–III subcylindrical, thickening apically; segment IV fusiform, apex pale. Postocular margins: dentiform, not exceeding outer eye margins.

Pronotum. Collar narrow; anterolateral angles angularly produced beyond collar; lateral margins sinuate medially, subparallel posteriorly; posterior margin broadly arcuate. Scutellum: triangular, with prominent median longitudinal carina. Male-specific characters: sternite VII with paired smooth median tubercles; pygophore cordate, dorsally with Y-shaped impression.

Abdomen. Connexivum: posterior margins of segments and lateral margin of segment VII with pale maculae. Spiracle placement: ventral on segments II–VII; spiracle VIII sublateral, faintly visible dorsally.

Etymology. The generic name is composed of “para-“ (Greek) close to and *Yangiella*.

Distribution. China (Yunnan)

 

#### 3.1.2. *Parayangiella latiovatusa* Bai, Cai & Heiss, sp. n. (Figure 1)

Type material. Holotype: male: China, Yunnan, Lushui, Sanhe Village, 19. V. 2023, Jia, Z.C.; Paratypes: 68 males, 71 females, same as above.

Description. Body: length 6.78 mm; surface densely granulate.

Head: anterior process extending to apex of antennal segment I; genae distinctly exceeding clypeus, converging anteriorly; clypeus convex. Antennae: 4-segmented; segments I and II subequal; IV longer than II; III longest (I:II:III:IV = 0.44:0.45:0.58:0.52); length/head width ratio 1.86; postocular denticles distinct, not exceeding outer eye margins.

Pronotum: subtrapezoidal; collar thin; anterolateral angles exceeding anterior collar margin; disk anteriorly flattened medially, irregularly depressed laterally with sublateral longitudinal sulci; posterior portion broad, flattened. Scutellum: well-developed, triangular; lateral margins carinate, subparallel; apex open; disk with irregular short transverse carinae. Hemelytra: Membrane extending to mid-tergum VII. Sterna III–VI: surface rugose with irregular longitudinal carinae.

Abdomen. Male genitalia: sternite VIII with reduced lateral lobes, not reaching mid-pygophore; pygophore well-developed, coarsely granulate; tergum with shallow depression. Spiracles: Ventral.

Measurements [in mm, ♀ (*n* = 3)/♂ (*n* = 3), holotype in parentheses]. Body length: 6.50–7.59/6.12–6.90 (6.78); Body width: 2.61–3.34/2.78–3.20 (3.11); Head length: 1.32–1.45/1.21–1.30 (1.28); Head width: 1.15–1.20/1.07–1.11 (1.10); Pronotum length: 1.14–1.45/1.15–1.31 (1.27); Pronotum width: 2.00–2.28/2.10–2.29 (2.26); Scutellum length: 0.99–1.24/0.97–1.10 (1.07); Scutellum width: 1.29–1.65/1.39–1.48 (1.45); Pygophore length: 0.15–0.21/0.56–0.60 (0.59); Pygophore width: 0.44–0.45/1.03–1.05 (1.05); Length antennal segments (I:II:III:IV): 0.41–0.45:0.42–0.45:0.54–0.61:0.52–0.54/0.41–0.45:0.43–0.46:0.55–0.60:0.50–0.53 (0.44:0.45:0.58:0.52).

Etymology. The specific epithet is a Latin compound adjective: latus (“broad”) and ovatus (“ovoid”), referring to the broadly ovoid abdominal dilation in dorsal view.

Distribution. Yunnan (Lushui)

**Figure 1:**
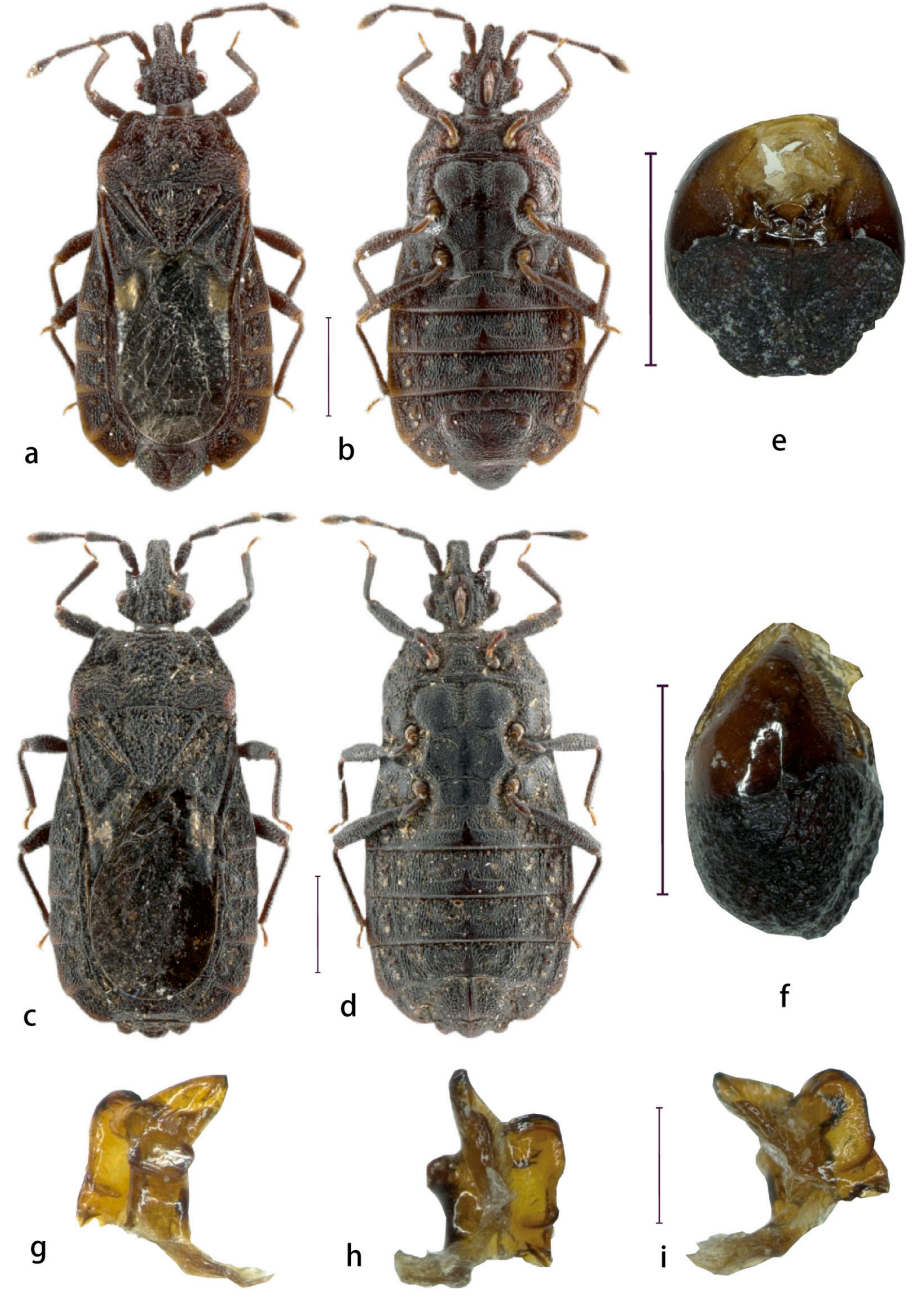
*Parayangiella latiovatusa* sp. n. Dorsal view of male holotype (**a**). Ventral view of male holotype (**b**). Dorsal view of female holotype (**c**). Ventral view of female holotype (**d**). The pygophore is shown in dorsal view (**e**). The pygophore is shown in lateral view (**f**). Right parameter (**g**–**i**). Scale bars = 1 mm for (**a**–**d**), 0.5 mm for (**e**,**f**), and 0.1 mm for (**g**–**i**).

### 3.2. Phylogenetic Position of Parayangiella

**Figure 2:**
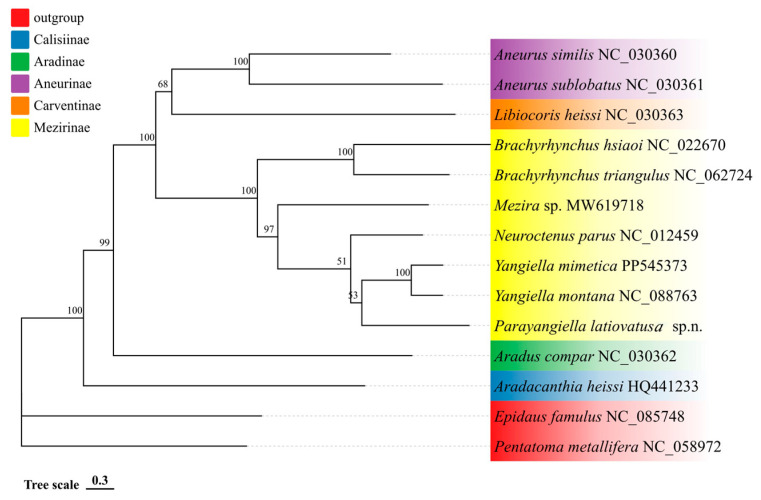
Phylogenetic tree of 16 species of Hemiptera inferred from the PCGsRNA data matrix using maximum likelihood (ML). Their support values are reported above the nodes. Different color blocks represent the various superfamilies within Pentatomomorpha. The subject of this study is *Parayangiella latiovatusa* sp. n.

**Figure 3:**
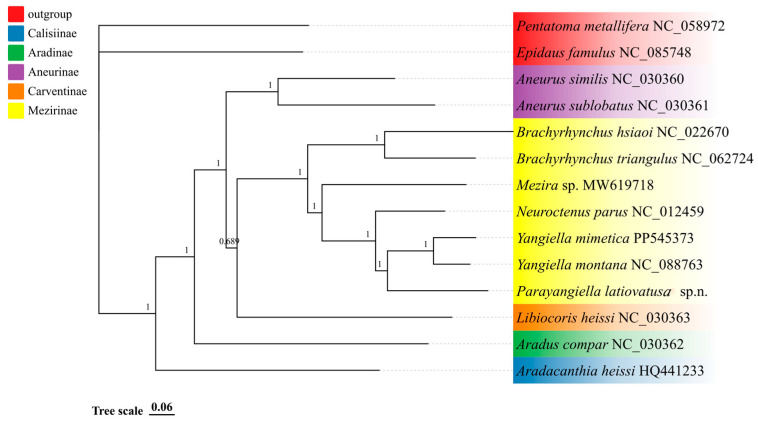
Phylogenetic tree of 16 species of Hemiptera inferred from the PCGsRNA data matrix using Bayesian inference (BI). Their support values are reported above the nodes. Different color blocks represent the various superfamilies within Pentatomomorpha. The subject of this study is *Parayangiella latiovatusa* sp. n.

In this study, phylogenetic analyses incorporating 12 species of Aradidae, with *Pentatoma metallifera* and *Epidaus famulus* as outgroups. The topological structures of both trees (Figure 2 and Figure 3) are congruent. The analysis recovered Calisiinae as the sister group to the remaining subfamilies. Within the main clade, Aradinae was resolved as sister to the clade comprising Carventinae, Aneurinae, and Mezirinae. Furthermore, Mezirinae is a highly evolved and monophyletic group, which is sister to a clade comprising Carventinae and Aneurinae. In Mezirinae, Brachyrhynchus is monophyletic. *Parayangiella latiovatusa* sp. n. forms a sister group with *Yangiella*.

### 3.3. Mitochondrial Structure of Parayangiella latiovatusa sp. n.

#### 3.3.1. Basic Characteristics of Mitochondrial Genes of *Parayangiella latiovatusa*

**Figure 4:**
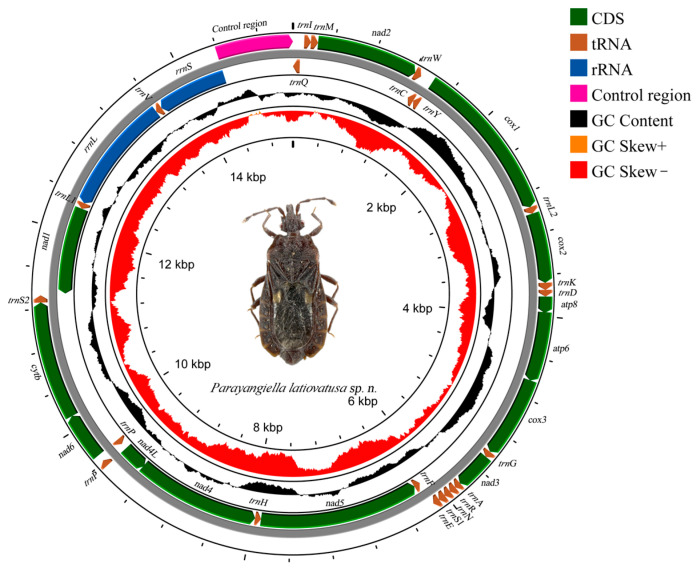
Mitochondrial genome structure map of *Parayangiella latiovatusa* sp. n.

The mitochondrial genome of *Parayangiella latiovatusa* sp. n., comprises 15,129 bp, features a closed circular double-stranded structure. Annotation revealed 23 genes encoded on the heavy strand (H-strand) and 14 genes on the light strand (L-strand) (Figure 4). The mitogenome exhibits a positive AT-skews, and the bias at the 1st and 2nd codons is lower than that at the 3rd codon (Table A4).

#### 3.3.2. Analysis of Protein-Coding Genes and Codon Usage

**Figure 5:**
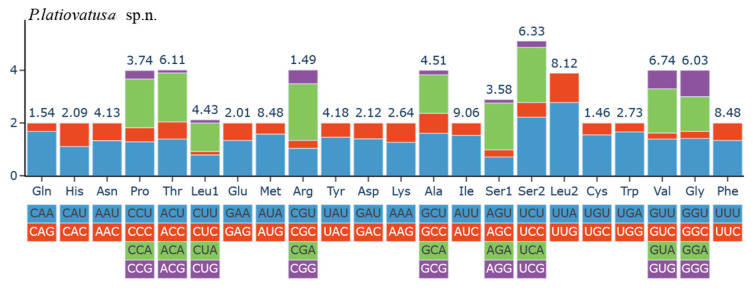
Relative synonymous codon usage of 13 protein-coding genes of *Parayangiella latiovatusa* sp. n. The numbers above the bar graph indicate the frequency of amino acids. The RSCU values are color-coded based on the codons below the amino acid labels.

Analysis of the 13 PCGs in *Parayangiella latiovatusa* sp. n. revealed their relative codon usage frequencies. The most common codons were AUU (Ile), AUA (Met), UUA (Leu), and UUU (Phe), whereas CGC (Arg), AGG (Ser), and ACG (Thr) occurred at markedly lower frequencies, indicating a clear amino-acid usage bias (Figure 5).

#### 3.3.3. Analysis of Base Substitution Saturation

**Figure 6:**
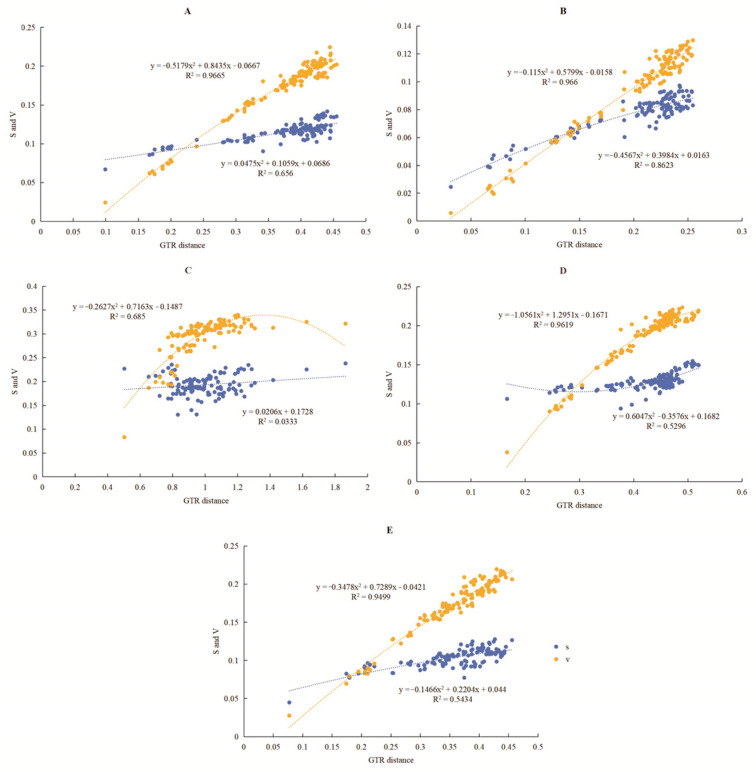
Substitution saturation plots for PCGs in the mt genomes of 14 Aradidae species. “S” signifies the transition rate (blue), and “V” signifies the transversion rate (yellow). (**A**) Protein-coding gene first site. (**B**) Protein-coding gene second site. (**C**) The third site of protein-coding genes. (**D**) All sites of protein-coding genes. (**E**) rRNA gene.

In both data matrices, the transversion rate within Aradidae frequently exceeds the transition rate, a pattern that aligns more closely with an ideal substitution state (Figure 6). Statistical analysis further supports this observation, yielding the *p*-value of less than 0.05 and the Iss value significantly lower than the Iss.cSym value. These results indicate an absence of substitution saturation in the nucleotide sequences.

#### 3.3.4. Ka/Ks Analysis of Protein-Coding Genes in Mitochondrial Genomes

**Figure 7:**
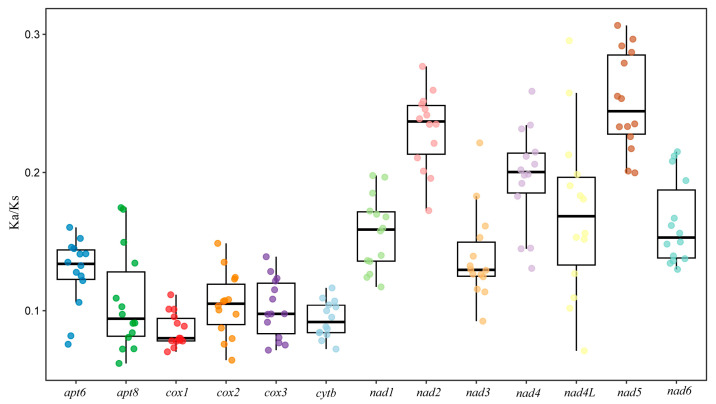
Nucleotide diversity and the ratio of Ka to Ks for PCGs in the mitochondrial genomes of 14 species belonging to Aradidae.

In this study, the evolutionary rates of the 13 protein-coding genes (PCGs) in Aradidae were quantified through Ka/Ks ratio analysis (Figure 7). It was found that the Ka/Ks ratio of all genes was below 1, signifying that these genes were under purifying selection and their protein functions were relatively stable. Among the protein-coding genes, nad5, nad2 and nad4 exhibited relatively high Ka/Ks values, suggesting elevated evolutionary rates, while cox1 had the lowest ratio, indicating it is the most conserved.

## 4. Discussion

The new species *Parayangiella latiovatusa* sp. n. conforms to the generic diagnosis of *Parayangiella*. While morphological analysis is fundamental to insect taxonomy, it can be insufficient for defining phylogenetic relationships. Therefore, we sequenced and characterized the mitochondrial genome of *Parayangiella latiovatusa* to elucidate its composition and structure.

The mitochondrial genome of the newly described *Parayangiella latiovatusa* sp. n. is consistent with the standard insect mitogenome in structure and exhibits a significant AT bias. This bias can be attributed to two main factors: (1) Mutation pressure and a lack of effective repair mechanisms during mitochondrial DNA replication lead to the accumulation of AT base pairs. (2) The compact structure and high gene density of the mitochondrial genome limit the increase in CG content. Genomes with high AT content are more conducive to the efficiency of replication and transcription, suggesting that the bias may represent a functional optimization of these genes [38,39]. Among the 22 tRNAs, we identified U-G, U-U, and A-C mismatches, which are likely influenced by the overall high AT content prevalent in insect mitochondria.

For all 13 protein-coding genes (PCGs), their Ka/Ks ratios were all below 1, indicating the presence of purifying selection. This selective pressure maintains the stability of gene functions by eliminating harmful mutations [40]. Substitution saturation analysis revealed that the nucleotide sequences of mitochondrial PCGs in Aradidae were unsaturated, thereby validating the suitability of the data for subsequent phylogenetic analyses [41]. Both phylogenetic trees of Aradidae, constructed based on 13 PCGs, indicated that *Parayangiella* gen. n. and *Yangiella* form a sister group. This result is highly consistent with the morphological classification, further verifying the taxonomic status of *Parayangiella* gen. n.

In summary, through morphological and molecular phylogenetic analyses of *Parayangiella latiovatusa* sp. n., its taxonomic status and evolutionary relationships have been clarified. Future studies need to incorporate larger sample sizes and more extensive genomic data to comprehensively understand the evolution and diversity of Aradidae.

## Data Availability

The genomic data in this study is available under the NCBI accession numbers PP737162.

## Appendix A

**Table A1:** Taxonomic information and GenBank accession numbers of mitogenomes used in this study.

Superfamily	Family	Subfamily	Species	Length	NCBI No.
Harpactorinae	Reduviidae	Harpactorinae	*Epidaus famulus*	16,135	NC 085748
Pentatominae	Pentatomidae	Pentatomoidea	*Pentatoma metallifera*	15,740	NC 058972
Aradoidea	Aradidae	Calisiinae	*Aradacanthia heissi*	15,528	HQ441233
		Aradinae	*Aradus compar*	16,814	NC 030362
		Aneurinae	*Aneurus similis*	16,477	NC 030360
		Aneurinae	*Aneurus sublobatus*	16,091	NC 030361
		Carventinae	*Libiocoris heissi*	15,168	NC 030363
		Mezirinae	*Brachyrhynchus hsiaoi*	15,250	NC 022670
		Mezirinae	*Brachyrhynchus triangulus*	15,170	NC 062724
		Mezirinae	*Mezira* sp. nov.	15,283	MW619718
		Mezirinae	*Neuroctenus parus*	15,354	NC 012459
		Mezirinae	*Yangiella mimetica*	15,192	PP545373
		Mezirinae	*Yangiella montana*	15,205	NC 088763
		Mezirinae	*Parayangiella latiovatusa* sp. nov.	15,129	PP737162

**Table A2:** Optimal partitioning strategy and evolution model of PCGsRNA IQ.

Subset	Name	Best Model
1	atp6 codon1 + atp8 codon1 + nad2 codon1	TIM + F + I + G4
2	atp6 codon2 + cox1 codon2 + cox2 codon2 + cox3 codon2 + cytb codon2	TVM + F + I + G4
3	atp6 codon3 + cox2 codon3 + cox3 codon3 + nad3 codon3	TPM3u + F + I + G4
4	atp8 codon2 + nad2 codon2 + nad3 codon2 + nad6 codon2	TVM + F + I + G4
5	atp8 codon3 + nad2 codon3	TPM3u + F + R2
6	cox1 codon1 + cox2 codon1 + cox3 codon1 + cytb codon1	GTR + F + I + I + R3
7	cox1 codon3 + cytb codon3	HKY + F + I + I + R4
8	nad1 codon1 + nad4L codon1 + nad4 codon1 + rrnL + rrnS	TVM + F + I + G4
9	nad1 codon2 + nad4L codon2 + nad4 codon2 + nad5 codon2	GTR + F + I + G4
10	nad1 codon3	HKY + F + G4
11	nad3 codon1 + nad6 codon1	TIM + F + I + G4
12	nad4L codon3	TN + F + G4
13	nad4 codon3	HKY + F + R3
14	nad5 codon1	TPM2u + F + I + G4
15	nad5 codon3	HKY + F + G4
16	nad6 codon3	HKY + F + R2

**Table A3:** Optimal partitioning strategy and evolution model of PCGsRNA BI.

Subset	Name	Best Model
1	atp6 codon1 + atp8 codon1 + nad2 codon1	GTR + F + I + G4
2	atp6 codon2 + cox1 codon2 + cox2 codon2 + cox3 codon2 + cytb codon2	GTR + F + I + G4
3	atp6 codon3 + cox2 codon3 + cox3 codon3 + nad3 codon3	HKY + F + I + G4
4	atp8 codon2 + nad2 codon2 + nad3 codon2 + nad6 codon2	GTR + F + I + G4
5	atp8 codon3 + nad2 codon3	HKY + F + G4
6	cox1 codon1 + cox2 codon1 + cox3 codon1 + cytb codon1	GTR + F + I + G4
7	cox1 codon3 + cytb codon3	GTR + F + I + G4
8	nad1 codon1 + nad4L codon1 + nad4 codon1 + rrnL + rrnS	GTR + F + I + G4
9	nad1 codon2 + nad4L codon2 + nad4 codon2 + nad5 codon2	GTR + F + I + G4
10	nad1 codon3	HKY + F + G4
11	nad3 codon1 + nad6 codon1	GTR + F + I + G4
12	nad4L codon3	HKY + F + G4
13	nad4 codon3	HKY + F + G4
14	nad5 codon1	HKY + F + I + G4
15	nad5 codon3	HKY + F + G4
16	nad6 codon3	HKY + F + G4

**Table A4:** Base composition and skewness of mitogenome of *Parayangiella latiovatusa* sp. n.

Location	Total/bp	A/%	T/%	G/%	C/%	A+T/%	AT-Skew	GC-Skew
Whole mitochondrial Genome (mtDNA)	15,129	40.4	28.5	12.5	18.5	68.9	0.172	−0.193
Protein-coding gene (PCGs)	10,929	30.3	38	15.8	15.9	68.3	−0.112	−0.005
1st codon	3643	33.9	31.5	21.4	13.3	65.4	0.037	0.234
2nd codon	3643	18.9	45.2	15.2	20.6	64.1	−0.409	−0.151
3rd codon	3643	38.1	37.3	10.7	13.9	75.4	0.011	−0.13
tRNAs	1442	36.6	35.7	15.3	12.3	72.3	0.012	0.108
rRNAs	1991	28.7	42	18.3	11	70.7	−0.188	0.249
CR	737	37	30.5	15.3	17.1	67.5	0.096	−0.054
